# Preoperative Diaphragmatic Excursion and Postoperative Pulmonary Complications After Laparoscopic Sleeve Gastrectomy: A Retrospective Cohort Study

**DOI:** 10.3390/medicina62102008

**Published:** 2026-10-17

**Authors:** Burcu Yormaz, Serdar Yormaz

**Affiliations:** 1 Department of Pulmonology, Faculty of Medicine, Selçuk University, 42250 Konya, Türkiye; 2 Department of Surgery, Faculty of Medicine, Selçuk University, 42250 Konya, Türkiye; serdaryormaz@selcuk.edu.tr

**Keywords:** bariatric surgery, sleeve gastrectomy, diaphragm ultrasonography, diaphragmatic excursion, postoperative pulmonary complications, ARISCAT, perioperative risk assessment

## Abstract

*Background and Objectives*: Postoperative pulmonary complications (PPCs) remain clinically relevant after bariatric surgery, while conventional risk assessment provides limited direct information on diaphragmatic performance. This study evaluated whether preoperative diaphragmatic excursion measured by ultrasonography was associated with European Perioperative Clinical Outcome (EPCO)-defined PPCs after laparoscopic sleeve gastrectomy (LSG) and whether it provided complementary discriminatory information beyond the Assess Respiratory Risk in Surgical Patients in Catalonia (ARISCAT) score. *Materials and Methods*: This retrospective observational cohort study included 128 adults who underwent primary elective LSG between May 2021 and March 2026. Preoperative bilateral diaphragmatic excursion was assessed by M-mode ultrasonography during quiet breathing and deep inspiration. The primary endpoint was occurrence of at least one EPCO-defined PPC during the index hospitalization. Associations were evaluated using parsimonious multivariable logistic regression, and discrimination was assessed using receiver operating characteristic analysis. *Results*: Seventy patients (54.7%) developed at least one PPC. Right deep inspiratory excursion showed the largest between-group difference (6.70 ± 0.4 cm in PPC-positive vs. 8.10 ± 0.6 cm in PPC-negative patients; *p* < 0.001). In the extended model, greater right deep inspiratory excursion was associated with lower odds of PPCs (adjusted OR per 1-cm increase 0.60, 95% CI 0.41–0.87; *p* = 0.007), while ARISCAT remained associated with PPC occurrence. ARISCAT alone yielded an area under the receiver operating characteristic curve (AUC) of 0.70 (95% CI 0.61–0.79), compared with 0.73 (95% CI 0.64–0.87) for right deep inspiratory excursion and 0.80 (95% CI 0.71–0.88) for the combined model. The combined model showed greater discrimination than ARISCAT alone (DeLong *p* = 0.03). In a sensitivity analysis excluding isolated minor atelectasis from the composite endpoint, the association remained directionally consistent but was no longer statistically significant (adjusted OR 0.75, 95% CI 0.48–1.02; *p* = 0.07). *Conclusions*: Lower preoperative diaphragmatic excursion was associated with EPCO-defined PPCs after LSG. Right deep inspiratory excursion may provide complementary physiological information to established clinical risk assessment, although prospective validation is required before routine clinical implementation.

## 1. Introduction

Obesity is associated with significant changes in respiratory physiology that may increase susceptibility to perioperative pulmonary dysfunction. Excess adipose tissue, particularly in the abdominal and thoracic compartments, increases the mechanical load on the respiratory system and leads to a decrease in chest wall compliance, functional residual capacity, expiratory reserve volume, and diaphragmatic mobility [1,2,3]. These abnormalities can lead to small airway closure—particularly in the supine position—ventilation–perfusion mismatch, and a reduction in respiratory reserve. This resulting mechanical disadvantage may become more pronounced during general anesthesia and laparoscopic surgery; in these situations, neuromuscular blockade, pneumoperitoneum, postoperative pain, and impaired cough reflex can further negatively affect ventilation and lung expansion [4]. These considerations are particularly important for patients undergoing laparoscopic bariatric procedures, where obesity-related respiratory dysfunction coincides with procedure-induced physiological stress.

Postoperative pulmonary complications (PPCs) continue to be a major cause of morbidity, prolonged hospital stays, and increased use of healthcare resources following major surgical procedures [4]. Therefore, the accurate preoperative identification of patients at high pulmonary risk is a critical component of perioperative assessment. Various clinical prediction tools have been developed for this purpose. Among these, the Assessment of Respiratory Risk in Surgical Patients in Catalonia (ARISCAT) score is one of the most extensively studied and has undergone a prospective external validation study [5,6]. However, clinical risk scores are primarily based on demographic, clinical, and procedure-related variables and do not directly and quantitatively measure dynamic respiratory muscle performance. Furthermore, a recent systematic review of PPC prediction models highlighted ongoing limitations regarding predictive performance and generalizability across different surgical populations [7]. Consequently, physiological measurements that provide complementary information to established clinical risk assessment may hold potential value.

The diaphragm is the primary muscle of inspiration and plays a central role in ensuring adequate ventilation, particularly when respiratory mechanics are impaired. Diaphragmatic ultrasound offers a non-invasive, radiation-free, bedside, and reproducible method for assessing diaphragmatic function. M-mode ultrasonography enables quantitative measurement of diaphragmatic movement during quiet tidal breathing and maximum inspiration; standardized methodologies and reference values have been established for this purpose [8,9,10]. Importantly, diaphragmatic movement represents a dynamic physiological parameter that is not directly captured by traditional perioperative clinical risk scores. Assessment during deep inspiration may be particularly informative, as maximum inspiratory effort places a greater functional demand on the diaphragm and may reveal limitations in inspiratory reserve that are less apparent during quiet breathing.

A growing body of perioperative evidence indicates that diaphragmatic dysfunction is associated with postoperative respiratory morbidity. However, substantial heterogeneity exists among published studies regarding the surgical populations studied, timing of ultrasonographic assessment, respiratory maneuvers performed, and ultrasonographic parameters evaluated [11,12,13,14,15]. Furthermore, a significant portion of the current literature has focused on postoperative diaphragmatic dysfunction. Measurements obtained postoperatively may reflect not only the patient’s underlying respiratory reserve but also the effects of anesthesia, mechanical ventilation, pneumoperitoneum, surgical trauma, and postoperative pain. In contrast, preoperative ultrasonography may offer the opportunity to characterize baseline diaphragmatic performance before these perioperative effects manifest. Moreover, evidence specifically addressing the relationship between preoperative diaphragmatic movement and PPCs in a homogeneous population undergoing laparoscopic sleeve gastrectomy (LSG) remains limited.

Consequently, this study investigated whether preoperative bilateral diaphragmatic movement measured during quiet breathing and deep inspiration in patients undergoing elective LSG was associated with PPCs as defined by the European Perioperative Clinical Outcomes (EPCO). Additionally, we evaluated whether diaphragmatic movement measured on the right side during deep inspiration provides complementary discriminatory information when assessed in conjunction with the ARISCAT score. Our objective was to test the hypothesis that lower preoperative diaphragmatic movement is associated with an increased likelihood of PPCs and that incorporating this physiological measurement could enhance discriminatory power beyond traditional clinical risk assessment.

## 2. Materials and Methods

### 2.1. Study Design and Participants

This retrospective observational cohort study was conducted in the Department of General Surgery at Selçuk University Faculty of Medicine, a tertiary referral center with an established bariatric surgery program. Consecutive adults undergoing primary elective LSG between May 2021 and March 2026 were screened. The study was reported in accordance with the Strengthening the Reporting of Observational Studies in Epidemiology (STROBE) statement [16].

No a priori sample-size or power calculation was performed because this retrospective study used all consecutive eligible patients with complete required data during the predefined study period. The final sample size was therefore determined by the number of patients meeting the eligibility criteria between May 2021 and March 2026.

Patients were eligible if they were aged ≥18 years, underwent primary elective LSG, had a body mass index > 35 kg/m^2^, completed standardized preoperative diaphragm ultrasonography, had a complete perioperative clinical dataset, and had postoperative pulmonary follow-up until discharge. Exclusion criteria were previous bariatric or upper gastrointestinal surgery, known diaphragmatic paralysis or a neuromuscular disorder affecting respiratory function, conversion to open surgery, emergency surgery, incomplete diaphragm ultrasonography, or missing postoperative outcome data.

**Figure 1:**
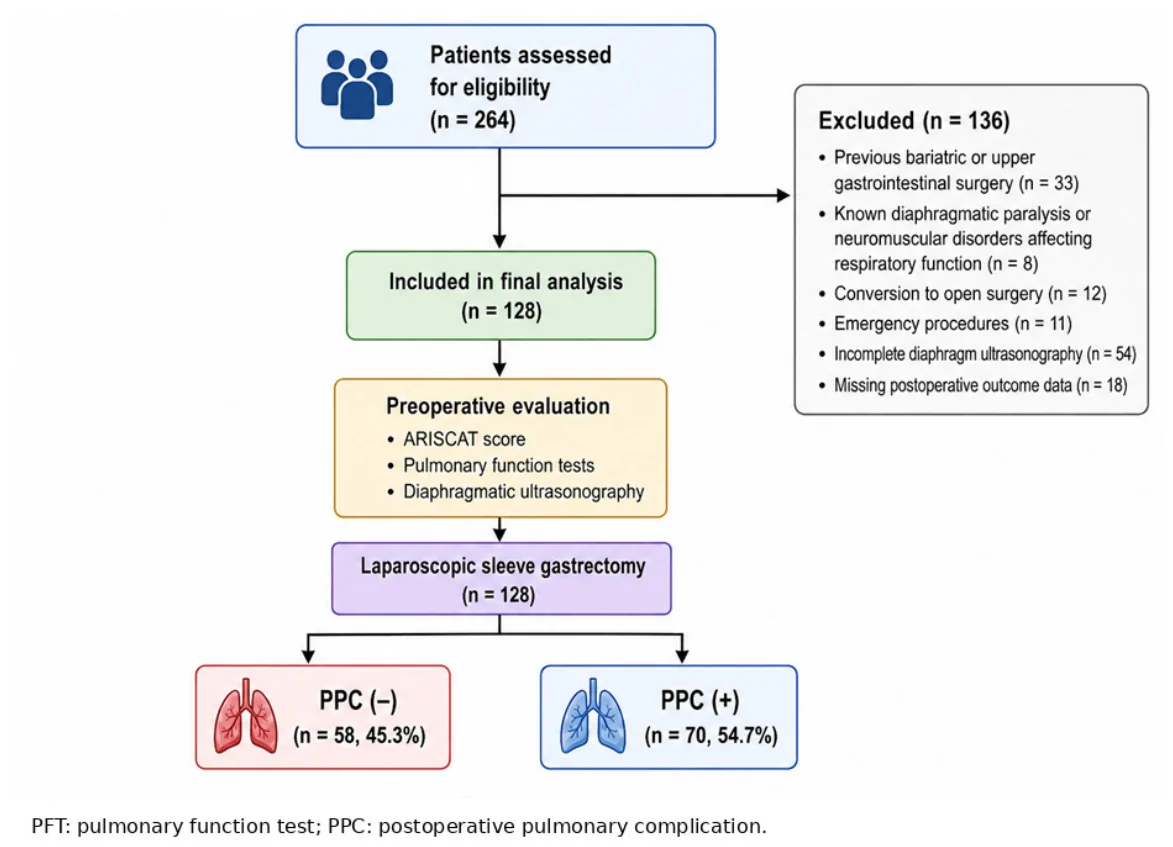
Study design and patient flow diagram. Of 264 patients assessed for eligibility, 136 were excluded, leaving 128 patients in the final analysis (PPC-negative, *n* = 58; PPC-positive, *n* = 70).

Among 264 patients assessed for eligibility, 136 were excluded for the following reasons: previous bariatric or upper gastrointestinal surgery (*n* = 33), known diaphragmatic paralysis or neuromuscular disorders affecting respiratory function (*n* = 8), conversion to open surgery (*n* = 12), emergency procedures (*n* = 11), incomplete diaphragm ultrasonography (*n* = 54), and missing postoperative outcome data (*n* = 18). The final cohort comprised 128 patients (Figure 1).

### 2.2. Preoperative Clinical Assessment

Preoperative evaluation followed the institutional bariatric surgery pathway and included demographic and anthropometric characteristics, comorbidities, laboratory testing, resting oxygen saturation, pulmonary function testing, and ARISCAT scoring. ARISCAT was analyzed as a continuous variable in the regression models [5,6].

### 2.3. Diaphragm Ultrasonography

All ultrasonographic examinations were performed preoperatively by the same board-certified general surgeon with formal point-of-care ultrasonography training. A BK2000 ultrasound system (BK Medical, Burlington, MA, USA) equipped with a 3.5–5.0 MHz convex transducer was used for ultrasonography. Diaphragm ultrasonography was not part of the routine standard preoperative assessment for all LSG patients. The measurements analyzed in this retrospective study had been obtained preoperatively as part of non-invasive educational/POCUS practice and did not alter diagnostic, surgical, or perioperative management decisions. Patients were examined in the supine position while breathing spontaneously. The right hemidiaphragm was assessed through a subcostal approach using the liver as an acoustic window, whereas the left hemidiaphragm was evaluated through the corresponding approach using the spleen when technically feasible. All measurements were obtained during the preoperative assessment before the index operation; the exact interval between ultrasonography and surgery was not consistently documented in the retrospective records.

Diaphragmatic excursion was quantified by M-mode ultrasonography during quiet tidal breathing and maximal voluntary inspiration. Patients were instructed to perform a maximal voluntary inspiration from resting expiration, and measurements were repeated when the respiratory maneuver or M-mode tracing was technically unsatisfactory. Four measurements were analyzed: right quiet-breathing excursion, right deep-inspiratory excursion, left quiet-breathing excursion, and left deep-inspiratory excursion. At least three technically satisfactory measurements were obtained for each maneuver and averaged for analysis, in accordance with established diaphragm-ultrasound methodology [8,9]. Diaphragm thickness and thickening fraction were not routinely available and were therefore not included in the present analysis.

### 2.4. Perioperative Management

Patients were managed within a standardized institutional perioperative pathway. Anesthetic management, laparoscopic surgical technique, postoperative mobilization, respiratory physiotherapy, and discharge criteria remained consistent throughout the study period. Contemporary evidence supports the importance of lung-protective perioperative ventilation in abdominal surgery and in patients with obesity [17,18].

### 2.5. Outcome Definitions

The primary outcome was the occurrence of at least one postoperative pulmonary complication during the index hospitalization, classified according to European Perioperative Clinical Outcome (EPCO) definitions [19]. Qualifying events included respiratory infection/pneumonia, respiratory failure, atelectasis, pleural effusion, pneumothorax, bronchospasm, and aspiration pneumonitis when the corresponding clinical criteria were met. For the binary primary endpoint, each patient contributed one PPC-positive or PPC-negative outcome regardless of the number of component events.

Because atelectasis accounted for a substantial proportion of the composite endpoint, we performed an exploratory sensitivity analysis using a more restrictive outcome definition. Forty-three patients with isolated minor atelectasis were reclassified as non-PPC, whereas clinically significant atelectasis or any other EPCO-defined PPC remained classified as PPC-positive.

### 2.6. Statistical Analysis

Statistical analyses were performed using IBM SPSS Statistics version 30.0 (IBM Corp., Armonk, NY, USA). Distributional assumptions for continuous variables were assessed using graphical methods and the Shapiro–Wilk test. Normally distributed variables are presented as mean ± standard deviation (SD), non-normally distributed variables as median (interquartile range), and categorical variables as *n* (%). Between-group comparisons were performed using the independent-samples *t* test or Mann–Whitney U test for continuous variables, as appropriate, and the chi-square test or Fisher’s exact test for categorical variables. Between-group *p* values in the baseline tables represent conventional descriptive group comparisons; no formal equivalence testing was performed.

Associations between preoperative diaphragmatic excursion measurements and PPC occurrence were first examined using univariable analyses. Between-group differences are reported with 95% confidence intervals (CIs), together with standardized effect sizes for the principal ultrasonographic comparisons. To reduce the risk of model overfitting, we used a parsimonious multivariable logistic regression strategy. ARISCAT served as the clinical reference model, and right deep inspiratory excursion was carried forward as the principal ultrasonographic variable after showing the strongest univariable association. Adjusted odds ratios (ORs) are reported with 95% CIs.

In the extended logistic regression model, ARISCAT score and right deep inspiratory diaphragmatic excursion were entered simultaneously as continuous variables. Thus, the adjusted odds ratio for diaphragmatic excursion represents its association with PPC occurrence conditional on ARISCAT score, whereas the adjusted ARISCAT estimate represents its association conditional on diaphragmatic excursion. The extended model was intentionally limited to these two predictors to evaluate the incremental contribution of the ultrasonographic parameter while minimizing model complexity and the risk of overfitting.

Model discrimination was assessed using receiver operating characteristic (ROC) analysis and the area under the curve (AUC) with 95% CI. Correlated AUCs derived from the same patients were compared pairwise using DeLong’s method [20], with ARISCAT serving as the reference model. ROC analyses were considered exploratory. The primary regression analyses were performed as complete-case analyses. PPC status, ARISCAT score, and right deep inspiratory excursion were complete for all 128 patients included in the analytic cohort; therefore, no imputation was required for variables entering the primary models. Calibration analysis was not performed. All tests were two-sided, and *p* < 0.05 was considered statistically significant.

## 3. Results

### 3.1. Study Population

A total of 128 patients were included in the final analysis. Seventy patients (54.7%) developed at least one EPCO-defined PPC, whereas 58 (45.3%) did not develop any EPCO-defined PPC (Figure 1). Baseline demographic and clinical characteristics are shown in Table 1, preoperative laboratory and respiratory functional variables in Table 2, diaphragmatic excursion measurements in Table 3, and operative/perioperative characteristics in Table 4. The distribution of individual EPCO-defined PPC components is provided in Supplementary Table S1.

### 3.2. Preoperative Diaphragmatic Excursion

**Figure 2:**
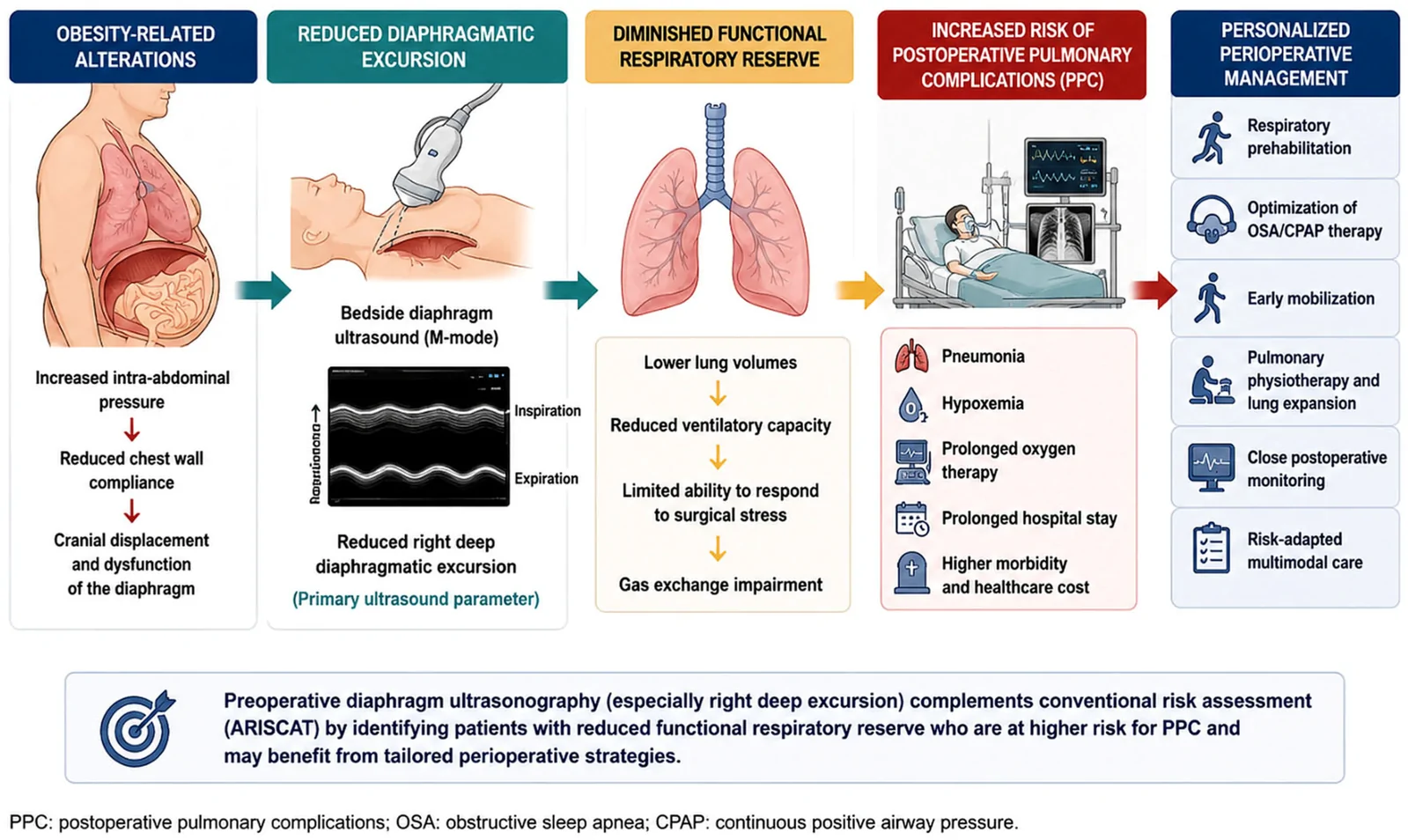
Central illustration of the proposed physiological framework linking reduced preoperative diaphragmatic excursion with reduced respiratory reserve and postoperative pulmonary vulnerability. The clinical management concepts shown are contextual and were not tested as interventions in this retrospective study.

All four excursion measurements were lower in the PPC-positive group (Table 3). The largest between-group difference was observed for right deep inspiratory excursion (6.70 ± 0.4 vs. 8.10 ± 0.6 cm; mean difference 1.40 cm, 95% CI 1.217–1.583; Cohen’s d = 2.80; *p* < 0.001). Right quiet-breathing excursion was 1.30 ± 0.2 versus 1.50 ± 0.4 cm (mean difference 0.20 cm, 95% CI 0.085–0.315; *p* < 0.001). Left quiet-breathing excursion was 1.25 ± 0.4 versus 1.70 ± 0.6 cm (mean difference 0.45 cm, 95% CI 0.267–0.633; *p* < 0.001), and left deep-inspiratory excursion was 6.36 ± 0.6 versus 7.20 ± 1.4 cm (mean difference 0.84 cm, 95% CI 0.447–1.233; *p* < 0.001). Right deep inspiratory excursion demonstrated the strongest univariable association with PPC occurrence and was therefore carried forward into the exploratory parsimonious multivariable model. A conceptual overview of the physiological framework linking to reduced diaphragmatic excursion with postoperative pulmonary vulnerability is provided in Figure 2.

### 3.3. Operative and Perioperative Characteristics

Operative duration, anesthesia duration, pneumoperitoneum duration, and intra-abdominal insufflation pressure did not differ significantly between groups (Table 4). Estimated blood loss was greater in the PPC-positive group (*p* = 0.007). Peak airway pressure, PEEP, and driving pressure were also higher in PPC-positive patients (all *p* < 0.001), who required longer postoperative oxygen supplementation and had longer hospital stays (both *p* < 0.001).

### 3.4. Multivariable Logistic Regression

In the reference model, ARISCAT was associated with PPC occurrence (OR 1.50, 95% CI 1.33–1.71; *p* < 0.001; Table 5). In the extended model, ARISCAT remained associated with PPCs (adjusted OR 1.42, 95% CI 1.24–1.62; *p* < 0.001), while greater right deep inspiratory excursion was associated with lower odds of PPC occurrence (adjusted OR per 1-cm increase 0.60, 95% CI 0.41–0.87; *p* = 0.007).

### 3.5. Discriminatory Performance and Sensitivity Analysis

**Figure 3:**
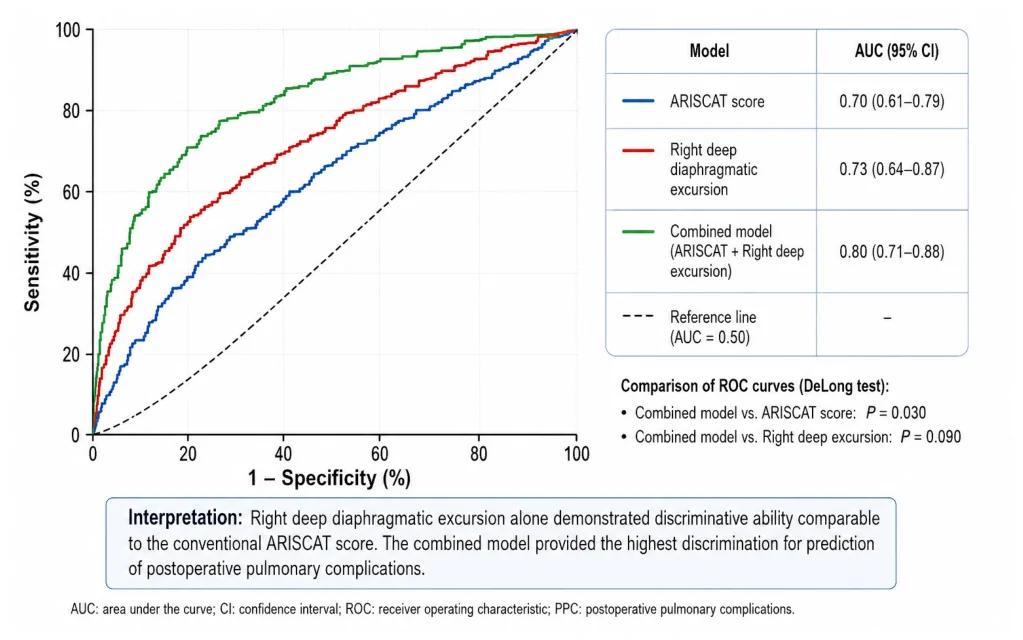
Receiver operating characteristic curves for discrimination of EPCO-defined postoperative pulmonary complications. ARISCAT: AUC 0.70 (95% CI 0.61–0.79); right deep inspiratory diaphragmatic excursion: AUC 0.73 (95% CI 0.64–0.87); combined model: AUC 0.80 (95% CI 0.71–0.88). DeLong comparisons: right deep excursion vs. ARISCAT, *p* = 0.09; combined model vs ARISCAT, *p* = 0.03.

As shown in Figure 3, ARISCAT demonstrated an AUC of 0.70 (95% CI 0.61–0.79), while right deep inspiratory excursion yielded an AUC of 0.73 (95% CI 0.64–0.87); this difference was not statistically significant (DeLong *p* = 0.09). The extended model achieved an AUC of 0.80 (95% CI 0.71–0.88), which was higher than that of ARISCAT alone (DeLong *p* = 0.03). In the sensitivity analysis, 43 patients with isolated minor atelectasis were reclassified as non-PPC, leaving 27 PPC-positive patients (21.1%). The association between right deep inspiratory excursion and PPCs remained directionally consistent but was attenuated after adjustment (adjusted OR 0.75, 95% CI 0.48–1.02; *p* = 0.07) (Table 6).

ROC curves showed the discriminatory performance of ARISCAT (AUC 0.70, 95% CI 0.61–0.79), right deep inspiratory diaphragmatic excursion (AUC 0.73, 95% CI 0.64–0.87), and the combined model (AUC 0.80, 95% CI 0.71–0.88). DeLong comparisons showed *p* = 0.09 for right deep inspiratory excursion versus ARISCAT and *p* = 0.03 for the combined model versus ARISCAT. ROC analyses were exploratory.

In the sensitivity analysis, 43 patients with isolated minor atelectasis were reclassified as non-PPC, leaving 27 PPC-positive (21.1%) and 101 PPC-negative (78.9%) patients. The association of right deep inspiratory excursion with the restricted endpoint remained directionally consistent but did not reach conventional statistical significance (adjusted OR 0.75, 95% CI 0.48–1.02; *p* = 0.07). Right deep inspiratory excursion retained good discrimination (AUC 0.82, 95% CI 0.67–0.97), and the extended model achieved an AUC of 0.84 (95% CI 0.75–0.93). The extended-versus-ARISCAT comparison did not reach statistical significance (DeLong *p* = 0.06) (Supplementary Table S2).

**Figure 4:**
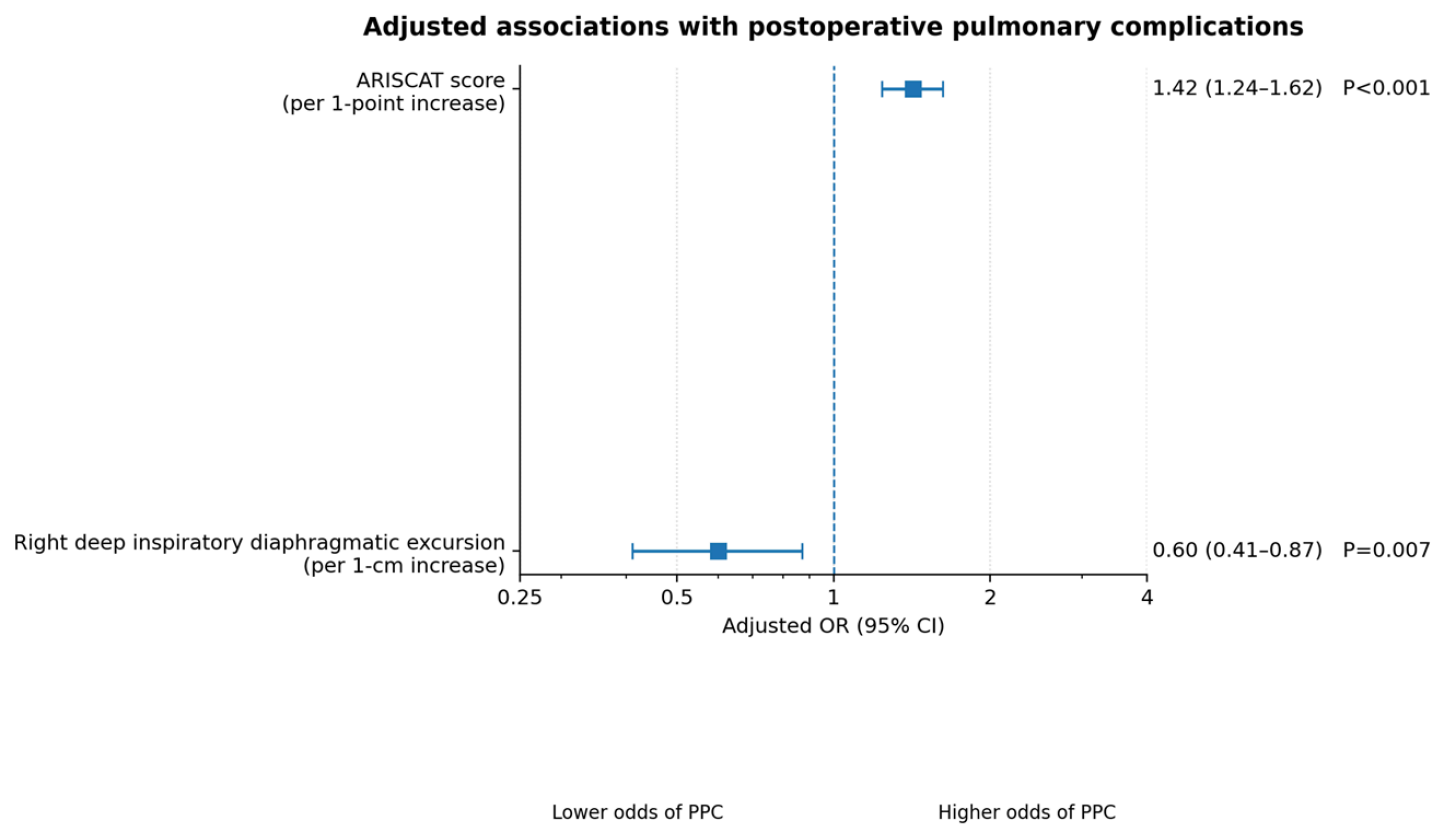
Forest plot of the final parsimonious multivariable logistic regression model. Adjusted odds ratios and 95% confidence intervals are shown for ARISCAT (adjusted OR 1.42, 95% CI 1.24–1.62; *p* < 0.001) and right deep inspiratory diaphragmatic excursion (adjusted OR per 1-cm increase 0.60, 95% CI 0.41–0.87; *p* = 0.007); the vertical reference line denotes an odds ratio of 1.0.

For visual comparison, the adjusted effect estimates from the final parsimonious multivariable logistic regression model are summarized in Figure 4.

## 4. Discussion

In this retrospective cohort of patients undergoing elective LSG, lower preoperative diaphragmatic excursion was associated with EPCO-defined PPCs. The clearest separation between groups was observed for right deep inspiratory excursion, which remained associated with PPC occurrence after adjustment for ARISCAT. The adjusted associations are summarized in Figure 4, and the extended model also showed greater discrimination than ARISCAT alone. These findings support diaphragm ultrasonography as a complementary physiological assessment rather than a replacement for established clinical risk stratification.

In this context, “complementary” should not be interpreted as evidence of established clinical utility. Rather, it indicates that right deep inspiratory excursion captured physiological information that remained associated with PPC occurrence after accounting for ARISCAT and was accompanied by improved discrimination in this cohort. Confirmation of incremental predictive value requires prospective validation incorporating calibration and clinical-utility analyses.

The observed association is physiologically plausible in patients with severe obesity. Increased abdominal mass and intra-abdominal pressure can alter diaphragmatic position and respiratory mechanics, while reductions in functional residual capacity and chest-wall compliance increase the mechanical burden on the respiratory system [1,2,3]. Anesthesia, pneumoperitoneum, postoperative pain, and ventilatory factors may further reduce lung volumes, thereby contributing to atelectasis and PPCs [4,5,6,7]. Diaphragmatic excursion may therefore reflect an aspect of functional respiratory reserve that is not fully captured by conventional clinical risk variables.

Conceptual illustration of the proposed relationship between obesity-related respiratory mechanical impairment, reduced diaphragmatic excursion, diminished functional respiratory reserve, and postoperative pulmonary complications. The clinical-management elements shown are conceptual and were not evaluated as interventions in the present study.

The greater separation observed during deep inspiration is noteworthy. Maximal inspiration imposes a greater functional demand on the inspiratory system than quiet tidal breathing and may reveal functional differences that are less apparent at rest. Previous ultrasound studies have demonstrated that excursion varies substantially according to respiratory maneuver and support standardized M-mode assessment [8,9,10].

The sensitivity analysis provides important context because atelectasis accounted for a substantial proportion of the composite endpoint. After isolated minor atelectasis was reclassified, the effect estimate remained directionally consistent, although the confidence interval crossed unity. The reduction in the number of events from 70 to 27 materially reduced statistical precision; therefore, this analysis should be interpreted as supportive rather than confirmatory.

Our findings are consistent with an expanding perioperative diaphragm-ultrasound literature. Vanamail et al. demonstrated an association between perioperative diaphragmatic inspiratory amplitude and PPCs after upper abdominal surgery [11]. Yan et al. subsequently reported that a postoperative maximal-inspiratory composite diaphragm index was associated with PPCs and that its addition to ARISCAT improved discriminatory performance [12]. Postoperative diaphragmatic dysfunction has also been associated with PPCs in esophageal and thoracic surgical cohorts [13,14], while a recent meta-analysis reported approximately threefold higher odds of PPCs among patients with ultrasound-detected postoperative diaphragmatic dysfunction [15].

The present study is methodologically distinct because ultrasonography was performed preoperatively and the cohort consisted exclusively of patients undergoing elective LSG. This temporal distinction shifts the potential role of ultrasound from detecting postoperative dysfunction to characterizing baseline vulnerability. Bariatric-specific studies have also shown that perioperative interventions can influence diaphragmatic excursion and respiratory function, reinforcing the relevance of this physiological parameter in patients with severe obesity [21,22,23].

The ARISCAT comparison should therefore be interpreted strictly as an exploratory incremental-discrimination analysis rather than as development or validation of a new prediction model. Although the extended model showed a higher AUC than ARISCAT alone, discrimination does not establish calibration, net clinical benefit, or readiness for implementation. Calibration was not assessed in the present retrospective analysis, and the model should therefore not be used for individual risk prediction. Prospective validation incorporating calibration, internal validation, decision-curve analysis, and external validation is required before any clinical implementation. This caution is consistent with broader reviews of PPC prediction models [7,24].

This study has several strengths. Diaphragm ultrasonography was performed using a standardized acquisition protocol, the same ultrasound system, and a single trained examiner, thereby reducing technical heterogeneity. Bilateral diaphragmatic excursion was assessed during both quiet breathing and deep inspiration, postoperative outcomes were classified according to EPCO definitions [19], and the cohort was restricted to a relatively homogeneous population undergoing elective laparoscopic sleeve gastrectomy. The analytical strategy was deliberately parsimonious, with ARISCAT serving as the clinical reference model and right deep inspiratory excursion evaluated as the principal physiological variable.

Several limitations should be acknowledged. First, the retrospective, single-center design limits causal inference and generalizability and leaves the analysis vulnerable to residual and unmeasured confounding. Second, all ultrasound examinations were performed by a single experienced operator; although this reduced interoperator variability during data acquisition, interobserver reproducibility could not be assessed. Third, diaphragm thickness and thickening fraction were not routinely available and therefore could not be incorporated into the analysis. Maximal inspiratory pressure (MIP) was also not systematically available; consequently, diaphragmatic excursion should be interpreted as a measure of diaphragmatic displacement during the respiratory maneuver rather than as a direct measurement of inspiratory muscle strength [10]. Fourth, the principal ultrasonographic predictor was selected after univariable comparison, and the incremental ARISCAT-plus-ultrasound model was exploratory; consequently, the observed improvement in discrimination may be optimistic. Fifth, model calibration and decision-curve analysis were not performed, and no internal resampling or external validation cohort was available. Sixth, atelectasis accounted for a substantial proportion of the primary composite endpoint. Although the sensitivity analysis excluding 43 patients with isolated minor atelectasis preserved the direction of the association, only 27 PPC events remained and the adjusted association no longer met the conventional threshold for statistical significance (*p* = 0.07), substantially reducing precision and limiting the complexity of the multivariable model. Although the extended primary model contained only two predictors for 70 PPC events, thereby limiting model complexity, right deep inspiratory excursion was selected after univariable comparison and no internal resampling was performed; therefore, some optimism in the estimated effect and discrimination remains possible. In addition, exclusion of patients with incomplete diaphragm ultrasonography or missing postoperative outcome data may have introduced selection bias, and the final analytic cohort may not fully represent all patients undergoing LSG.

Finally, the study was not designed to establish a clinically actionable threshold for diaphragmatic excursion. Prospective multicenter studies incorporating standardized acquisition, formal intra- and interobserver reproducibility assessment, diaphragm thickness and thickening fraction, direct respiratory-muscle strength measures such as MIP, prespecified evaluation of right–left diaphragmatic differences, calibration, clinical-utility analyses, and external validation are needed before routine implementation.

## 5. Conclusions

Preoperative diaphragmatic excursion was associated with EPCO-defined PPCs after laparoscopic sleeve gastrectomy, with the greatest between-group difference observed for right deep inspiratory excursion. When considered alongside ARISCAT, diaphragmatic excursion appeared to provide complementary physiological information relevant to perioperative pulmonary risk assessment. Prospective multicenter studies incorporating standardized acquisition, reproducibility assessment, prespecified modelling, calibration assessment, clinical-utility analysis, and external validation are needed before routine clinical implementation.

## Figures and Tables

**Table 1:** Baseline demographic and clinical characteristics.

Variable	Overall Cohort	PPC (−)	PPC (+)	*p* Value
Number of patients, *n*	128	58 (45.3%)	70 (54.7%)	
Age, years	39.11 ± 10.6	36.21 ± 8.6	41.52 ± 8.6	<0.001
Sex, male, *n* (%)	74	25	49	0.004
Height, cm	168 ± 15.24	170 ± 8.48	166 ± 10.27	0.017
Weight, kg	116 ± 22.41	115 ± 20.11	118 ± 10.46	0.308
BMI, kg/m^2^	40.83 ± 3.42	39.3 ± 2.58	42.1 ± 5.16	<0.001
Smoking status, *n* (%)				0.019
Never smoked	54 (42.1%)	32 (55.1%)	22 (31.4%)	--
Current smoking, *n* (%)	35 (27.4%)	14 (24.2%)	21 (30.2%)	--
Former smoking, *n* (%)	39 (30.5%)	12 (20.7%)	27 (38.6%)	--
Hypertension, *n* (%)	72 (56.3%)	26 (44.8%)	46 (65.7%)	0.028
Diabetes mellitus, *n* (%)	65 (50.8%)	12 (20.6%)	53 (75.7%)	<0.001
Obstructive sleep apnea, *n* (%)	76 (59.4%)	26 (44.8%)	50 (71.4%)	0.004
COPD or asthma, *n* (%)	44 (34.3%)	10 (17.2%)	34 (48.5%)	<0.001
ASA physical status, *n* (%)				<0.001
ASA I-II	75 (58.6%)	44 (75.8%)	31 (44.3%)	
ASA III	53 (41.4%)	14 (24.1%)	39 (55.7%)	
ARISCAT score	34.97 ± 4.6	28.3 ± 6.7	40.5 ± 5.5	<0.001

Data are means ± SD or *n* (%), as appropriate. The *p* value for ASA physical status represents the omnibus between-group comparison. BMI, body mass index; COPD, chronic obstructive pulmonary disease; ASA, American Society of Anesthesiologists; ARISCAT, Assess Respiratory Risk in Surgical Patients in Catalonia.

**Table 2:** Preoperative laboratory and respiratory functional assessment.

Variable	Overall Cohort	PPC (−)	PPC (+)	*p* Value
Resting SpO_2_, %	92 ± 2.4	94 ± 1.6	90 ± 3.2	<0.001
Hemoglobin, g/dL	12.8 ± 1.5	11.75 ± 1.4	13.67 ± 1.6	<0.001
White blood cell count, ×10^9^/L	11.12 ± 3.4	9.55 ± 2.2	12.42 ± 4.6	<0.001
C-reactive protein, mg/L	18.83 ± 6.8	10.82 ± 5.7	25.47 ± 7.9	<0.001
Albumin, g/dL	3.3 ± 0.6	3.7 ± 0.7	2.9 ± 0.5	<0.001
FEV_1_, L	2.46 ± 0.6	2.35 ± 0.4	2.55 ± 0.8	0.070
FEV_1_, % predicted	81.3 ± 10.4	83.5 ± 9.4	79.2 ± 6.8	0.004
FVC, L	3.26 ± 0.6	3.25 ± 0.62	3.23 ± 0.74	0.868
FVC, % predicted	84.5 ± 8.5	83.9 ± 11.2	84.7 ± 5.1	0.617
FEV_1_/FVC, %	75.71 ± 12.28	76.42 ± 11.52	74.38 ± 12.45	0.338

Data are mean ± SD. SpO_2_, peripheral oxygen saturation; FEV_1_, forced expiratory volume in 1 s; FVC, forced vital capacity.

**Table 3:** Preoperative diaphragmatic excursion according to EPCO-defined PPC status.

Ultrasound Parameter (cm)	Overall (*n* = 128)	PPC (−) (*n* = 58)	PPC (+) (*n* = 70)	Difference (95% CI)	Cohen’s d	*p* Value
Right quiet breathing	1.39 ± 0.3	1.50 ± 0.4	1.30 ± 0.2	0.20 (0.085–0.315)	0.65	<0.001
Right deep inspiration	7.33 ± 0.8	8.10 ± 0.6	6.70 ± 0.4	1.40 (1.217–1.583)	2.80	<0.001
Left quiet breathing	1.45 ± 0.3	1.70 ± 0.6	1.25 ± 0.4	0.45 (0.267–0.633)	0.90	<0.001
Left deep inspiration	6.74 ± 0.9	7.20 ± 1.4	6.36 ± 0.6	0.84 (0.447–1.233)	0.81	<0.001

Values are mean ± SD. Differences are expressed as PPC (−) minus PPC (+). CI, confidence interval; EPCO, European Perioperative Clinical Outcome; PPC, postoperative pulmonary complication.

**Table 4:** Operative and perioperative characteristics.

Variable	Overall Cohort	PPC (−)	PPC (+)	*p* Value
Operative duration (min)	56.58 (44.15–72.15)	58.21 (44.16–64.47)	55.82 (49.26–85.68)	0.158
Anesthesia duration (min)	66.56 (54.15–78.27)	64.22 (57.19–71.38)	68.44 (62.11–74.76)	0.186
Pneumoperitoneum duration (min)	36.21 (27.15–47.22)	30.15 (27.19–40.09)	38.13 (32.24–40.15)	0.594
Intra-abdominal pressure (mmHg)	14.15 ± 2	14.04 ± 2	14.48 ± 3	0.324
Estimated blood loss (mL)	350.0 (130.0–460.0)	310.0 (195.0–340.0)	390.0 (270.0–415.5)	0.007
Intraoperative peak airway pressure (cmH_2_O)	34.60 ± 5.28	31.19 ± 9.26	37.42 ± 11.44	<0.001
Mean PEEP (cmH_2_O)	6 ± 0.7	5 ± 1.1	7 ± 0.9	<0.001
Mean driving pressure (cmH_2_O)	12.38 ± 3.8	10.9 ± 2.2	13.6 ± 3.4	<0.001
Postoperative oxygen duration (h)	0.6 ± 0.2	0.4 ± 0.2	0.8 ± 0.7	<0.001
Length of hospital stay (days)	4.6 ± 0.5	2.7 ± 0.3	6.5 ± 0.6	<0.001

Data are means ± SD or *n* (%), as appropriate. The *p* value for ASA physical status represents the omnibus between-group comparison. BMI, body mass index; COPD, chronic obstructive pulmonary disease; ASA, American Society of Anesthesiologists; ARISCAT, Assess Respiratory Risk in Surgical Patients in Catalonia.

**Table 5:** Parsimonious multivariable logistic regression models.

Model/Predictor	Adjusted OR (95% CI)	*p* Value
Model 1: ARISCAT only		
ARISCAT score (continuous)	1.50 (1.33–1.71)	<0.001
Model 2: ARISCAT + right deep inspiratory excursion		
ARISCAT score (continuous)	1.42 (1.24–1.62)	<0.001
Right deep inspiratory excursion (per 1-cm increase)	0.60 (0.41–0.87)	0.007

Note. OR, odds ratio; CI, confidence interval; ARISCAT, Assess Respiratory Risk in Surgical Patients in Catalonia. ARISCAT score and right deep inspiratory diaphragmatic excursion were entered as continuous variables. The extended model was intentionally restricted to these two predictors to provide a parsimonious assessment of the incremental contribution of diaphragmatic excursion beyond conventional clinical risk assessment. Model discrimination (AUC and DeLong comparisons) is reported separately in Table 6.

**Table 6:** Discriminatory performance of the final models.

Parameter/Model	AUC (95% CI)	DeLong *p* vs. ARISCAT
ARISCAT	0.70 (0.61–0.79)	-
Right deep inspiratory excursion	0.73 (0.64–0.87)	0.09
ARISCAT + right deep inspiratory excursion	0.80 (0.71–0.88)	0.03

## Data Availability

The data supporting the findings of this study are available from the corresponding author upon reasonable request. The data are not publicly available because they contain clinical information subject to institutional and ethical restrictions.

## Supplementary Materials

The following supporting information can be downloaded at https://www.mdpi.com/article/10.3390/medicina62102008/s1. Table S1: Distribution of EPCO-defined postoperative pulmonary complications; Table S2: Sensitivity analysis excluding isolated minor atelectasis from the composite PPC endpoint. These supplementary analyses provide additional detail on endpoint composition and robustness of the primary findings.

## Abbreviations

The following abbreviations are used in this manuscript:

ARISCAT	Assess Respiratory Risk in Surgical Patients in Catalonia
ASA	American Society of Anesthesiologists
AUC	Area under the receiver operating characteristic curve
BMI	Body mass index
CI	Confidence interval
DE	Diaphragmatic excursion
EPCO	European Perioperative Clinical Outcome
FEV_1_	Forced expiratory volume in 1 s
FVC	Forced vital capacity
LSG	Laparoscopic sleeve gastrectomy
OR	Odds ratio
PEEP	Positive end-expiratory pressure
PFT	Pulmonary function test
PPC	Postoperative pulmonary complication
ROC	Receiver operating characteristic
SD	Standard deviation
SpO_2_	Peripheral oxygen saturation
