# Supplementary material for: Preoperative Diaphragmatic Excursion and Postoperative Pulmonary Complications After Laparoscopic Sleeve Gastrectomy: A Retrospective Cohort Study
Source: Medicina (Kaunas). 2026 Oct 17;62(10):2008. doi: 10.3390/medicina62102008 (PMC13706973; doi:10.3390/medicina62102008)
Supplement: Supplementary file 1 [file medicina-62-02008-s001.zip › medicina-4577592-supplementary.pdf]

Supplementary Table S1. Distribution of EPCO-defined postoperative pulmonary complications.

PPC component	n /N(%)
Respiratory infection	2/128(1.6)
Respiratory failure	2/128(1.6)
Pleural effusion	6/128 (4.7)
Atelectasis	55/128(43.0)
of which isolated minor atelectasis	43/128(33.6)
Pneumothorax	1/128(0.8)
Bronchospasm	3/128(2.3)
Aspiration pneumonitis	1/128(0.8)
≥1 EPCO-defined PPC	70 /128(54.7)

Supplementary Table S2. Sensitivity analysis excluding isolated minor atelectasis from the composite PPC endpoint.

Parameter	Primary EPCO-defined PPC analysis	Sensitivity analysis excluding isolated minor atelectasis
PPC-positive, n/N (%)	70/128 (54.7%)	27/128 (21.1%)
PPC-negative, n/N (%)	58/128 (45.3%)	101/128 (78.9%)
ARISCAT, adjusted OR (95% CI)	1.42 (1.24–1.62)	1.26 (0.94–1.70)
Right deep inspiratory DE, adjusted OR per 1-cm increase (95% CI)	0.60 (0.41–0.87)	0.75 (0.48–1.02)
P value for right deep inspiratory DE	0.007	0.07
ARISCAT AUC (95% CI)	0.70 (0.61–0.79)	0.48 (0.25–0.68)
Right deep inspiratory DE AUC (95% CI)	0.73 (0.64–0.87)	0.82 (0.67–0.97)
ARISCAT + right deep inspiratory DE AUC (95% CI)	0.80 (0.71–0.88)	0.84 (0.75–0.93)
DeLong P, extended model vs ARISCAT	0.03	0.06
